# A Watch-Type Electrocardiography Is a Reliable Tool for Detecting Paroxysmal Cardiac Arrhythmias

**DOI:** 10.3390/jcm11123333

**Published:** 2022-06-10

**Authors:** Yun Gi Kim, Jong-Il Choi, Hee-Jung Kim, Kyongjin Min, Yun Young Choi, Jaemin Shim, Ho Sung Son, Young-Hoon Kim

**Affiliations:** 1 Department of Internal Medicine, Division of Cardiology, Korea University Anam Hospital, Korea University College of Medicine, Seoul 02841, Korea; tmod0176@gmail.com (Y.G.K.); mkj880628@naver.com (K.M.); yych60@naver.com (Y.Y.C.); jaemins@korea.ac.kr (J.S.); yhkmd@unitel.co.kr (Y.-H.K.); 2 Department of Thoracic & Cardiovascular Surgery, Korea University Anam Hospital, Korea University College of Medicine, Seoul 02841, Korea; heejung440@hanmail.net

**Keywords:** arrhythmia, wearable device, electrocardiography, smartwatch, watch-type ECG

## Abstract

**Background**: A substantial proportion of cardiac arrhythmias are paroxysmal in nature, and 12-lead electrocardiography (ECG) and Holter monitoring often fail to detect paroxysmal arrhythmias. We designed and evaluated a watch-type, electrocardiograph-recording, wearable device (w-ECG) to overcome the limitations of 12-lead ECG and Holter monitoring. **Methods**: We prospectively enrolled 96 patients with symptoms assumed to be related to cardiac arrhythmias. Electrocardiography recording was performed with both the w-ECG and Holter monitoring. Detection of any arrhythmia was the primary outcome endpoint and was compared between the w-ECG and Holter monitoring. **Results**: Any arrhythmia was detected in 51 (53.1%) and 27 (28.1%) patients by the w-ECG and Holter monitoring, respectively (odds ratio (OR) = 2.9, *p* < 0.001). The w-ECG was superior to Holter monitoring for the detection of clinically significant arrhythmias (excluding atrial premature contraction, ventricular premature contraction, and non-sustained atrial tachyarrhythmia) (OR = 2.34, *p* = 0.018). In 27 (28.1%) patients, cardiac arrhythmias were detected only by the w-ECG, with atrial fibrillation being the most frequent case (13 patients). Based on ECGs recorded by using the w-ECG, 17 patients (17.7%) received therapeutic interventions, including radiofrequency catheter ablation. **Conclusions**: The w-ECG is capable of recording ECGs of good quality, with a discernable P wave and distinguishable QRS morphology. The ability of the w-ECG to detect cardiac arrhythmias was significantly better than that of Holter monitoring, and a significant proportion of patients received therapeutic intervention based on ECGs recorded by the w-ECG.

## 1. Introduction

The global burden of cardiac arrhythmia is growing rapidly, mainly due to the increasing prevalence of atrial fibrillation (AF) [1,2,3]. Significant progress in the field of radiofrequency catheter ablation (RFCA) and cardiac implantable electronic devices has enabled effective treatment of many cardiac arrhythmias, including paroxysmal supraventricular tachycardia (PSVT), atrial tachycardia (AT), AF, ventricular tachycardia (VT), and cardiac conduction disorders [4,5,6,7]. Although some arrhythmias, such as persistent AF and complete atrioventricular nodal block, are easily detected, others are hard to diagnose, despite obvious patient symptoms. This is due to the paroxysmal nature of many arrhythmias. Unless electrocardiography (ECG) is performed at the time of ongoing arrhythmia, PSVT, AT, paroxysmal AF, and VT may not be diagnosed. Unfortunately, those paroxysmal arrhythmias might disappear on the way to the hospital for a 12-lead ECG evaluation. Holter monitoring might fail to detect paroxysmal arrhythmias if they do not occur on the day of examination. To overcome these limitations of 12-lead ECG and Holter monitoring, various wearable devices have been developed, including watch-type devices [8,9]. Tison et al. reported that smartwatch photoplethysmography can detect AF, albeit not as accurately as 12-lead ECG [8]. The Apple Heart study reported that 34% of participants who received an irregular pulse notification had atrial fibrillation upon subsequent ECG patch evaluation [9]. However, watch-type devices in the aforementioned studies used photoplethysmography, which only detects the pulse and not the electrical signals of the heart.

We designed a watch-type, wearable ECG device (w-ECG), called a Memowatch, to detect electrical signals from the heart and translate these into single-lead ECG recordings (HUINNO Co., Ltd., Seoul, Korea). Each recorded ECG was initially analyzed by an artificial intelligence-based algorithm, and only ECGs assumed to represent cardiac arrhythmia were presented to on-duty cardiologists. The diagnostic yield of the w-ECG was evaluated in this prospective study. Our goal was to compare the diagnostic capabilities of traditional Holter monitoring and the w-ECG to detect cardiac arrhythmias.

## 2. Methods

### 2.1. Participant Population

This prospective study was performed at Korea University Medicine Anam Hospital, Seoul, Korea. Patients were enrolled if (i) they had symptoms of an arrhythmia, such as palpitations, chest pain, or dizziness; (ii) 12-lead ECG failed to reveal any type of arrhythmia assumed to be the cause of symptoms; and (iii) they were older than 19 years and younger than 81 years. Exclusion criteria were as follows: (i) patients younger than 19 years or older than 81 years; (ii) inability to record ECG using the w-ECG, due to factors such as contact dermatitis or mental disability; (iii) contraindications to Holter monitoring or patient refusal to undergo Holter monitoring; (iv) inability to link the w-ECG to a personal smartphone. If the underlying arrhythmia responsible for the patient’s symptoms was obvious on 12-lead ECG or other diagnostic modalities, the patient was excluded from the study. Prior diagnosis of arrhythmia was not an exclusion criterion, and we enrolled such patients if (i) the arrhythmia was treated by ablation and had no documented recurrence, or (ii) the arrhythmia had been stable and no documented ECGs to explain the symptoms were available at the time of enrollment. Patient enrollment was performed at the Department of Cardiology and Cardiothoracic Surgery. The Institutional Review Board of Korea University Medicine Anam Hospital approved this study, and written informed consent was obtained from all patients. The study protocol strictly adhered to the ethical guidelines of the 2013 Declaration of Helsinki and the legal regulations of Korea.

### 2.2. Smartwatch for ECG Monitoring

**Figure 1:**
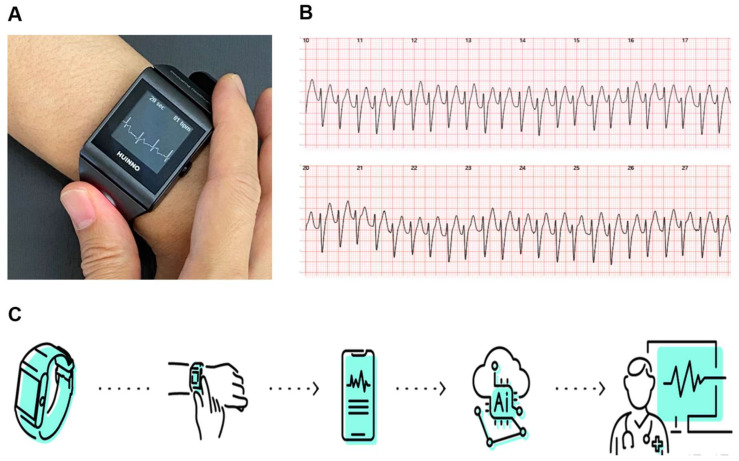
The watch-type ECG device (w-ECG): (**A**) Representative image of the w-ECG. (**B**) ECG recorded by the w-ECG. (**C**) Processing of the w-ECG. Recorded ECGs are sent to the centralized server and analyzed by an artificial intelligence-based algorithm. ECG: electrocardiography.

The w-ECG was developed by HUINNO, a medical device company in the Republic of Korea. The device is certified by the Ministry of Food and Drug Safety of Korea as a medical device to record ECGs. The w-ECG has two electrodes: one at the bottom of the watch body, and the other at the top of the wrist band. To start recording a 30 s ECG, the subject wearing the w-ECG ensures contact between the bottom electrode and their wrist and places their opposite finger on the top electrode. A representative image of the w-ECG is provided in Figure 1. After the 30 s ECG recording is finished, it is transferred to an interconnected mobile application, also developed by HUINNO, through Bluetooth Low Energy, followed by transfer to a cloud-based remote server that stores and analyzes the ECG. Physicians can monitor the uploaded ECGs.

### 2.3. Machine Learning

To develop an algorithm to analyze ECGs, we retrieved 10,592 single-lead ECGs (lead I). Data were labeled as normal sinus rhythm, AF, and other. We randomly divided the ECG dataset into two different groups: a training group and a validation group, at a 4:1 ratio. Residual network (ResNet) focuses only on the local part of the ECG, whereas SE-ResNet, which is a ResNet with a squeeze-and-excitation block, checks the overall ECG by considering the interaction between local parts of the ECG. Due to this characteristic, we used a 152-layer SE-ResNet that shows the highest accuracy when used to classify lead II ECG for the seven-class ECG classification [10]. The difference between the proposed network and the original network is that the proposed deep-learning model is a 3-class classification model for lead I ECG [10]. To optimize the model, we used the Adam optimizer with an initial learning rate of 0.001 and cross-entropy as the loss function. The training process continued until validation loss did not decrease for a certain step. Using the model created through this process, we evaluated lead I ECG data measured by using the w-ECG. 

### 2.4. Study Procedures and Outcome Endpoints

The final diagnosis of an ECG recorded by either the w-ECG or Holter monitoring was based on consensus among the researchers (Y.G. Kim, J.-I. Choi, and H.S. Son). If there was a discrepancy regarding diagnosis, the ECG was interpreted by another cardiologist and then adjudicated by a committee blinded to any diagnosis.

The occurrence of any arrhythmia was the primary outcome endpoint of this study, and was compared between the w-ECG and Holter monitoring. A major arrhythmia was defined as any clinically significant arrhythmia, except for atrial premature contraction (APC), ventricular premature contraction (VPC), or non-sustained AF, AT, or atrial flutter. The diagnostic yield of major arrhythmia was compared between the w-ECG and Holter monitoring.

### 2.5. Statistical Analysis

We used Student’s *t*-test to assess the significance of differences between continuous variables. Fisher’s exact test or the Chi-square test was used to assess the significance of differences between categorical variables. Odds ratios were calculated by logistic regression, with a fixed effect for the treatment group (w-ECG vs. Holter monitoring). All tests were two-tailed, and p values equal to, or less than, 0.05 were considered statistically significant. All statistical analyses were performed using SAS version 9.4 (SAS Institute, Cary, NC, USA).

## 3. Results

### 3.1. Patients

**Figure 2:**
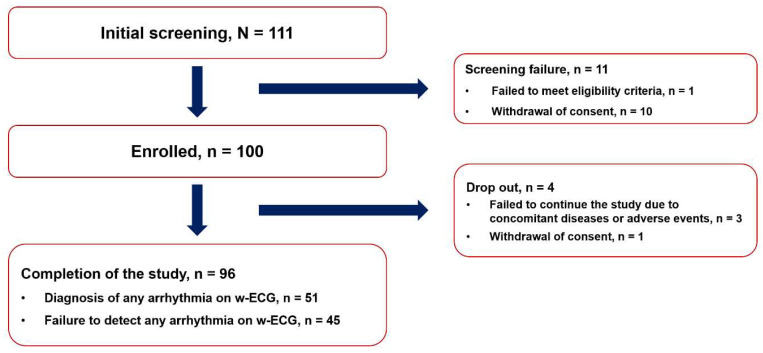
Study flow.

From March 2020 to August 2020, a total of 111 patients with symptoms of cardiac arrhythmia were screened for inclusion in this study. Ten patients withdrew their informed consent, and two patients failed to meet the eligibility criteria. Three patients failed to continue the study, due to concomitant disease or adverse events. Finally, 96 patients completed the study and were included in the final analysis (Figure 2).

Baseline demographics are summarized in Table 1. The mean age was 48.3 ± 14.4 years, and 51.0% of participants were male. Before study enrollment, 28 patients (29.2%), 14 patients (14.6%), and 1 (1.0%) patient had a prior diagnosis of AF, atrial flutter (typical or atypical), and PSVT, respectively. Three patients had a prior history of syncope, and two patients had a history of ischemic stroke. Holter monitoring was performed for one day for all participants. The mean duration of the w-ECG evaluation was 25.06 ± 6.74 days.

### 3.2. ECGs Acquired by Using the w-ECG

**Figure 3:**
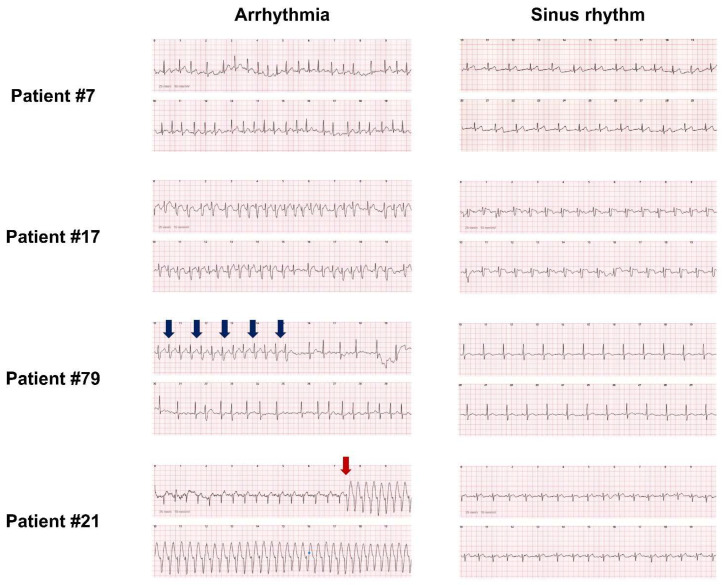
Atrial fibrillation and ventricular tachycardia detected by the w-ECG. Patients #7, #17, and #79 were diagnosed with atrial fibrillation based on w-ECG recordings. The w-ECG was able to record rate-dependent functional QRS widening in patient #79 (blue arrows). Patient #21 was diagnosed with ventricular tachycardia and the initiation point was clearly captured (red arrow). w-ECG: watch-type ECG device.

**Figure 4:**
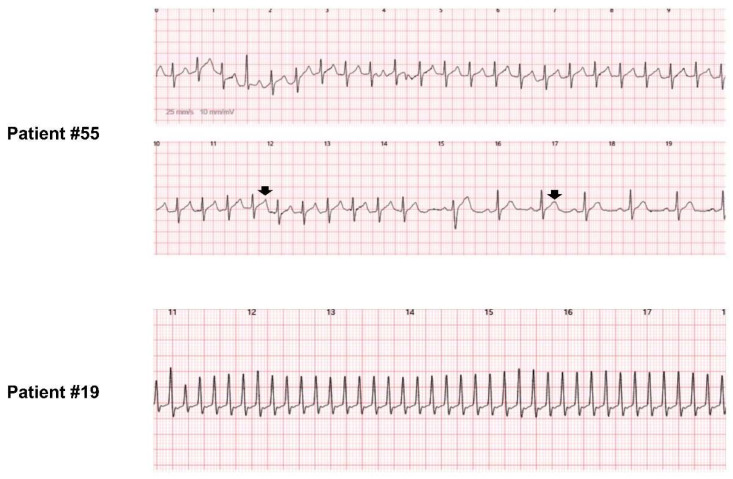
Paroxysmal supraventricular tachycardia and atrial tachycardia detected by the w-ECG. Patient #55 was diagnosed with paroxysmal supraventricular tachycardia using the w-ECG, and electrophysiology study revealed atrioventricular nodal reentrant tachycardia, fast–slow type. Different T-wave morphology during tachycardia and sinus rhythm (black arrows) is compatible with atrioventricular nodal reentrant tachycardia, fast–slow type. Sudden termination of tachycardia was clearly recorded, and the mode of termination was A-no-V, suggestive of paroxysmal supraventricular tachycardia. This image suggests that the w-ECG can detect subtle differences in T-wave morphology. Patient #19, with a prior history of radiofrequency catheter ablation for atrial fibrillation, was diagnosed with narrow QRS complex tachycardia using the w-ECG. Electrophysiology demonstrated that the tachycardia was atypical atrial flutter, and it was successfully ablated. w-ECG: watch-type ECG device.

Representative ECGs acquired by using the w-ECG are presented in Figure 3. The w-ECG was able to record the P wave (atrial activity) during sinus rhythm (right panel of Figure 3). Fibrillatory waves during AF were also detectable by the w-ECG (left panel of patients #7, #17, and #79 in Figure 3). Importantly, the w-ECG was able to discriminate QRS morphology. In patient #79, the initial QRS complex was widened, probably due to rate-dependent functional conduction delay; after a decrease in heart rate, the QRS interval narrowed (Figure 3). Initiation of VT was clearly visible in patient #21, based on QRS morphology (Figure 3). Patient #55 recorded not only ongoing paroxysmal supraventricular tachycardia but also the moment of termination (Figure 4). Patient #19, who had a prior radiofrequency catheter ablation for AF two years ago, recorded narrow QRS complex tachycardia with the w-ECG, which was found to be atypical atrial flutter during the electrophysiology study (Figure 4). These findings suggest that the w-ECG can provide crucial information not only with regard to heart rate and RR interval but also detection of P waves and QRS morphology discrimination, which are advantages compared to photoplethysmography-based wearable devices.

### 3.3. Detection of Arrhythmias

**Figure 5:**
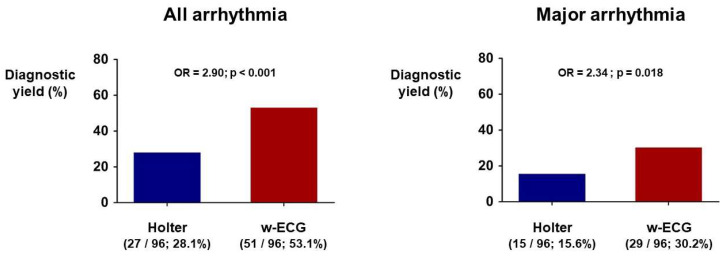
Diagnostic yield of Holter monitoring versus the w-ECG. The w-ECG was superior to Holter monitoring at detecting all arrhythmias and major arrhythmias. OR: odds ratio; w-ECG: watch-type ECG device.

The cardiac arrhythmias diagnosed by the w-ECG and Holter monitoring are summarized in Table 2. Among 96 patients who completed the study, any arrhythmia was detected in 51 (53.1%) and 27 (28.1%) patients in the w-ECG and Holter monitoring groups, respectively (Figure 5). The odds ratio for the probability of detecting any cardiac arrhythmia by the w-ECG, compared with Holter monitoring, was 2.9 (*p* < 0.001; Figure 5). The w-ECG was superior to Holter monitoring for the detection of major arrhythmias (excluding APC, VPC, non-sustained AF, and non-sustained atrial flutter) (OR = 2.34, *p* = 0.018; Figure 5). The w-ECG also showed a significantly better ability than Holter monitoring to detect AF (OR = 2.6, *p* = 0.017; Table 2).

In 27 (28.1%) patients, cardiac arrhythmias were detected only by the w-ECG (Table 3). The most frequent major arrhythmia detectable only by the w-ECG was AF (13 patients), followed by atrial flutter (3 patients), and VT (2 patients). Three atrial flutter events and one second-degree Mobitz type I atrioventricular block were detectable only by Holter monitoring. Two atrial flutters were asymptomatic, and one atrial flutter terminated before activation of the w-ECG by the patient. The second-degree Mobitz type I atrioventricular block was asymptomatic and could not be recorded by the w-ECG.

### 3.4. Therapeutic Interventions

A total of 14 patients underwent RFCA for a cardiac arrhythmia detected on the w-ECG: 10 patients, 2 patients, 1 patient, and 1 patient with AF, VT, atypical atrial flutter, and PSVT, respectively (Table 4). Two patients with AF did not undergo RFCA but were prescribed antiarrhythmic drugs, while one patient with AF did not undergo RCFA but was prescribed an anticoagulant. The number of w-ECG evaluations needed to have any therapeutic interventions or RFCAs was 5.65 (96/17) and 6.86 (96/14), respectively (Table 4).

Among eleven AF patients who underwent RFCA, AF was not detected by Holter monitoring in six of these patients. One atypical atrial flutter and two VT events eliminated by RFCA were not detected by Holter monitoring.

## 4. Discussion

The main findings of this study are as follows: (i) the w-ECG, a watch-type device, is capable of recording ECGs and detecting various cardiac arrhythmias, (ii) both the QRS complex and P wave are recordable by the w-ECG, (iii) the morphology of the QRS complex is discernable with the w-ECG, (iv) the w-ECG is superior to Holter monitoring at detecting cardiac arrhythmias, and (v) ECG evaluation by using the w-ECG resulted in therapeutic interventions in 17.7% of patients. The advantages of the w-ECG are that (i) it is a watch-type, wearable device, which maximizes patient convenience; (ii) it is ECG-based and not photoplethysmography-based; (iii) it can record P waves; and (iv) it can discriminate QRS morphology. Our study demonstrates the clinical usefulness of the w-ECG and also provides evidence that it is better than Holter monitoring at detecting cardiac arrhythmias.

### 4.1. Diagnostic Discrepancy in Clinical Practice: Paroxysmal Nature of Arrhythmias

In principle, a diagnosis of arrhythmia is confirmed by “symptom–rhythm correlation” when ECG-based rhythm status is recorded during ongoing symptoms. However, a large proportion of cardiac arrhythmias, such as paroxysmal AF or PSVT, are paroxysmal in nature. When ECGs are not taken at the time of the arrhythmia, they provide no useful information, and an accurate diagnosis is not possible. Implantable loop recorders are one potential solution. However, these devices are installed via an invasive procedure, and their cost-effectiveness is controversial. Although Holter monitoring or patch-type devices have been used to address the limitations of 12-lead ECG recordings, they are limited by the duration of evaluation: 1–2 days for Holter monitoring and 1–2 weeks for patch-type devices, respectively [11,12]. If a given arrhythmia occurs rarely but is highly symptomatic, it is essential to record the arrhythmia at the time of the event. Patient-activated ECG recording devices that are always available are the method of choice for evaluating such paroxysmal arrhythmias. Watch-type devices have an advantage over other types of devices, in that they are always available to record ECGs at the discretion of the patient. However, most watch-type devices are photoplethysmography-based and can only detect a pulse, not ECG [9]. Photoplethysmography-based devices, such as Fibricheck (Qompium, Hasselt, Belgium), or old versions of watch-type devices cannot detect electrical signals and have limited capabilities to discriminate between atrial and ventricular arrhythmias [13,14]. They can also underestimate actual heart rate when heart rate is rapid and irregular, such as AF with a rapid ventricular response. The w-ECG tested in this study (Memowatch) has a clear advantage over photoplethysmography-based devices, in that it can detect P waves and discriminate between morphologies of the QRS complex, a critical component of arrhythmia detection. Portable event recorders, such as the AliveCor device, including its latest version, KardiaMobile 6L (AliveCor Inc, Mountain View, CA, USA), can record ECGs, but their portability is significantly inferior to that of watch-type devices because they are not wearable [15,16]. The w-ECG has the potential to be widely used to diagnose paroxysmal arrhythmias based on its advantages in portability and the ability to record ECGs at the patient’s discretion.

### 4.2. Therapeutic Interventions

An accurate diagnosis of arrhythmia is important because therapeutic interventions are based on the diagnosis. Not all de novo diagnoses of cardiac arrhythmia lead to therapeutic interventions, but still, important clinical decisions can be made. A diagnosis of AF can lead to anticoagulation, if indicated, to prevent ischemic stroke, or RFCA to eliminate AF itself [17,18,19]. Detection of PSVT or VT can also lead to successful treatment of the disease via RFCA. In this study, a total of 17 patients (17.7%) had some type of therapeutic intervention based on ECGs recorded by the w-ECG. Among 17 patients, 14 underwent RFCA to treat cardiac arrhythmias, demonstrating that the w-ECG is capable of detecting paroxysmal-type arrhythmias, which can lead to definitive therapeutic interventions.

Up to 20% of all strokes are attributable to AF, and anticoagulation is an essential part of the management of patients with AF [20,21,22]. Non-paroxysmal AF is easily detected by Holter monitoring or 12-lead ECG. However, it may be challenging to diagnose paroxysmal AF through Holter monitoring or 12-lead ECG if the burden of AF is low. Detection of paroxysmal AF by a symptom-initiated ECG recording by using the w-ECG can have a profound impact on subsequent therapeutic interventions. In this study, 13 patients (13.5%) were diagnosed with AF only via the w-ECG. The poor prognosis of AF-related ischemic stroke and the fact that RFCA can significantly reduce AF burden and symptoms further emphasize the utility of the w-ECG [23,24].

### 4.3. Limitations

This study has several limitations. First, the w-ECG is not a continuous ECG monitoring device and requires activation by the patient. Therefore, asymptomatic arrhythmia events are likely to be missed by using the w-ECG. Arrhythmias causing dizziness are unlikely to be recorded by the w-ECG. Second, all patients enrolled in this study had symptoms of arrhythmia. Therefore, the clinical usefulness of the w-ECG in asymptomatic patient needs to be evaluated in future studies (e.g., AF screening). Third, the w-ECG has not yet been released commercially, and the cost of evaluation has not yet been determined; thus, we could not assess the cost-effectiveness of the w-ECG. Fourth, our cohort consisted exclusively of patients seen at a single center; therefore, it is unclear how generalizable our findings are. Finally, the quality of ECGs recorded by various watch-type ECG devices, such as Memowatch, Apple watch (Apple Inc., Cupertino, CA, USA), or Galaxy watch (Samsung Electronics, Seoul, Korea), needs to be compared with each other.

## 5. Conclusions

The w-ECG, an ECG-based, watch-type, wearable device, showed superior performance to Holter monitoring in detecting symptomatic cardiac arrhythmias. Discernable P waves were recorded by the w-ECG, and the morphology of the QRS complex was distinguishable. Major cardiac arrhythmias were detected by the w-ECG in 30.2% of patients, and 17.7% of patients underwent a therapeutic intervention based on the w-ECG recording.

## Figures and Tables

**Table 1:** Baseline demographics.

	N = 96
**Age (years)**	48.3 ± 14.4
**Sex**	
Male	49 (51.0%)
Female	47 (49.0%)
**Height (cm)**	166.7 ± 8.9
**Weight (kg)**	68.3 ± 13.6
**Cardiovascular disorders**	
Atrial fibrillation	28 (29.2%)
Atrial flutter (typical)	10 (10.4%)
Atrial flutter (atypical)	4 (4.2%)
Premature atrial contraction	7 (7.3%)
Premature ventricular contraction	9 (9.4%)
Paroxysmal supraventricular tachycardia	1 (1.0%)
Complete atrioventricular block	1 (1.0%)
Angina pectoris	4 (4.2%)
Variant angina	1 (1.0%)
Myocardial infarction	1 (1.0%)
**Hypertension**	26 (27.1%)
**Diabetes mellitus**	3 (3.1%)
**Dyslipidemia**	23 (24.0%)
**Depression**	2 (2.1%)
**Anxiety disorder**	3 (3.1%)
**Panic disorder**	1 (1.0%)
**Syncope**	3 (3.1%)
**Ischemic stroke**	2 (2.1%)
**Transient ischemic attack**	1 (1.0%)
**Echocardiographic findings**	
Left atrial dimension (mm)	36.1 ± 5.5
Left ventricular ejection fraction (%)	55.8 ± 4.4
Left ventricular end-diastolic dimension (mm)	46.7 ± 4.6
E/e’	8.0 ± 3.2
Severe valvular disease	0 (0%)
**Medications**	
Flecainide	9 (9.4%)
Propafenone	12 (12.5%)
Amiodarone	4 (4.2%)
Pilsicainide	2 (2.1%)
Bisoprolol	12 (12.5%)
Carvedilol	7 (7.3%)
Nebivolol	2 (2.1%)
Edoxaban	4 (4.2%)
Apixaban	3 (3.1%)
Dabigatran	1 (1.0%)
Warfarin	2 (2.1%)
Antiplatelets	10 (10.4%)

Severe valvular disease: mitral stenosis/regurgitation or aortic stenosis/regurgitation of a severe degree diagnosed by cardiologist.

**Table 2:** Detection of various arrhythmias by Holter monitoring and the w-ECG.

	Holter Monitoring (n = 96)	w-ECG (n = 96)
Atrial fibrillation	11 (11.5%)	24 (25.0%)
Non-sustained atrial fibrillation or atrial tachycardia	4 (4.2%)	2 (2.1%)
Atrial flutter (typical)	0 (0.0%)	0 (0.0%)
Atrial flutter (atypical)	3 (3.1%)	3 (3.1%)
Paroxysmal supraventricular tachycardia	1 (1.0%)	1 (1.0%)
Ventricular tachycardia	0 (0.0%)	2 (2.1%)
Non-sustained ventricular tachycardia	0 (0.0%)	1 (1.0%)
Atrial premature contraction	6 (6.3%)	8 (8.3%)
Ventricular premature contraction	6 (6.3%)	12 (15.5%)
Tachycardia-bradycardia syndrome	0 (0.0%)	1 (1.0%)
Sinus pause	0 (0.0%)	1 (1.0%)
Second degree atrioventricular block	1 (1.0%)	0 (0.0%)
Detection of any arrhythmia (patient number)	27 (28.1%)	51 (53.1%)
Detection of any arrhythmia (event number)	32	55
Detection of major arrhythmia * (patient number)	15 (15.6%)	29 (30.2%)
Detection of major arrhythmia * (event number)	16	31

* Excluding sinus pause, premature atrial contraction, premature ventricular contraction, non-sustained atrial tachycardia, and non-sustained atrial fibrillation. w-ECG: watch-type ECG device.

**Table 3:** Arrhythmias detected only by either the w-ECG or Holter monitoring.

	N = 96
**Arrhythmias detected only by the w-ECG (patient number)**	27 (28.1%)
**Arrhythmias detected only by the w-ECG (event number)**	32
Major arrhythmias * detected only by the w-ECG (event number)	19
Atrial fibrillation	13
Atrial flutter (typical or atypical)	3
Non-sustained atrial fibrillation or tachycardia	3
Ventricular tachycardia	2
Premature atrial contraction	4
Premature ventricular contraction	6
Tachycardia–bradycardia syndrome	1
**Arrhythmias detected only by Holter monitoring (patient number)**	3 (3.1%)
**Arrhythmias detected only by Holter monitoring (event number)**	8
Major arrhythmias * detected only by Holter monitoring (event number)	4
Atrial fibrillation	0
Atrial flutter (typical or atypical)	3
Non-sustained atrial fibrillation or tachycardia	2
Ventricular tachycardia	0
Premature atrial contraction	2
Premature ventricular contraction	0
Second-degree atrioventricular block	1

* Excluding sinus pause, premature atrial contraction, premature ventricular contraction, non-sustained atrial tachycardia, and non-sustained atrial fibrillation. w-ECG: watch-type ECG device.

**Table 4:** Detection of arrhythmias by the w-ECG that resulted in therapeutic interventions.

N	Detected Arrhythmia	Simultaneous Detection by Holter Monitoring	Treatment Modification
1	Atrial fibrillation	No	RFCA
2	Ventricular tachycardia	No	RFCA
3	Paroxysmal supraventricular tachycardia	Yes	RFCA
4	Atrial fibrillation	Yes	RFCA
5	Atrial fibrillation	Yes	Anticoagulation
6	Atrial fibrillation	Yes	RFCA
7	Atrial fibrillation	No	RFCA
8	Atrial fibrillation	No	RFCA
9	Atrial tachycardia	No	RFCA
10	Atrial fibrillation	Yes	RFCA
11	Atrial fibrillation	Yes	RFCA
12	Atrial fibrillation	No	Antiarrhythmic drugs
13	Atrial fibrillation	Yes	RFCA
14	Atrial fibrillation	No	RFCA
15	Atrial fibrillation	Yes	Antiarrhythmic drugs
16	Atrial fibrillation	No	RFCA
17	Ventricular tachycardia	No	RFCA

RFCA: radiofrequency catheter ablation; w-ECG: watch-type ECG device.

## Data Availability

Data can be made available by the corresponding author upon reasonable request.
